# Copy Neutral LOH Affecting the Entire Chromosome 6 Is a Frequent Mechanism of *HLA* Class I Alterations in Cancer

**DOI:** 10.3390/cancers13205046

**Published:** 2021-10-09

**Authors:** Maria Antonia Garrido, Francisco Perea, Jose Ramon Vilchez, Teresa Rodríguez, Per Anderson, Federico Garrido, Francisco Ruiz-Cabello, Natalia Aptsiauri

**Affiliations:** 1 Servicio de Radiología, UGC de Radiología, Hospital Virgen de la Nieves, 18014 Granada, Spain; mariaa.garrido.collado.sspa@juntadeandalucia.es; 2 Servicio de Análisis Clínicos e Inmunología, UGC de Laboratorio Clínico, Hospital Universitario Virgen de las Nieves, 18014 Granada, Spain; franciscoj.perea.garcia@juntadeandalucia.es (F.P.); joser.vilchez.sspa@juntadeandalucia.es (J.R.V.); teresa.rodriguez.ruiz.sspa@juntadeandalucia.es (T.R.); per.anderson@ibsgranada.es (P.A.); federico.garrido.sspa@juntadeandalucia.es (F.G.); 3 Instituto de Investigación Biosanitaria IBS.GRANADA, 18014 Granada, Spain; 4 Departamento de Bioquímica, Biología Molecular III e Inmunología, Facultad de Medicina, Universidad de Granada, 18071 Granada, Spain

**Keywords:** cancer immune escape, *HLA* class I, copy-neutral loss of heterozygosity (CN-LOH), antigen presentation, beta2-microglobulin, cytotoxic T-cells, cancer immunotherapy

## Abstract

**Simple Summary:**

Loss of antigen presentation due to the altered expression of tumor *HLA* class I (*HLA-I*) molecules is a common mechanism of cancer immune escape. Loss of *HLA-I* haplotype, locus or a single allele due to genetic and chromosomal aberrations, may result in reduced antigen presentation and, thus, facilitate immune evasion. Here, we demonstrate the prevalence of the copy neutral loss of heterozygosity (CN-LOH) involving *HLA-I* heavy chain and *B2M* gene in various human tumor cell lines and tissues. We discuss the impact of the LOH in *HLA* genes on tumor immune rejection, clonal expansion and association with the cancer recurrence in the immunotherapy settings. It represents a genetic barrier for effective treatment and can be considered as a potential genetic biomarker of cancer immune escape.

**Abstract:**

Total or partial loss of *HLA* class I antigens reduce the recognition of specific tumor peptides by cytotoxic T lymphocytes favoring cancer immune escape during natural tumor evolution. These alterations can be caused by genomic defects, such as loss of heterozygosity at chromosomes 6 and 15 (LOH-6 and LOH-15), where *HLA* class I genes are located. There is growing evidence indicating that LOH in *HLA* contributes to the immune selection of *HLA* loss variants and influences the resistance to immunotherapy. Nevertheless, the incidence and the mechanism of this chromosomal aberration involving *HLA* genes has not been systematically assessed in different types of tumors and often remains underestimated. Here, we used SNP arrays to investigate the incidence and patterns of LOH-6 and LOH-15 in a number of human cancer cell lines and tissues of different histological types. We observed that LOH in *HLA* is a common event in cancer samples with a prevalence of a copy neutral type of LOH (CN-LOH) that affects entire chromosome 6 or 15 and involves chromosomal duplications. LOH-6 was observed more often and was associated with homozygous *HLA* genotype and partial *HLA* loss of expression. We also discuss the immunologic and clinical implications of LOH in *HLA* on tumor clonal expansion and association with the cancer recurrence after treatment.

## 1. Introduction

Tumor cells grow and metastasize despite an active cellular immune response mediated by T lymphocytes that recognize tumor antigens as peptides presented by human leucocyte antigens class I molecules (*HLA-I*). *HLA* is a complex genetic system encoding three heavy chain proteins (*HLA-A*, *HLA-B* and *HLA-C*) that are associated at the cell surface with a light chain, i.e., the β2-microglobulin. *HLA*-I molecules present different antigenic peptides to T-cells and stimulate cellular immune responses. It is well known that loss of tumor *HLA-I* expression is a common mechanism of cancer immune escape from cytotoxic T-lymphocytes (CTL) [1]. There are multiple molecular mechanisms causing altered *HLA-I* expression in tumors, including mutations of *HLA-I* heavy chain and *β2-microglobulin* (*B2M*) genes [2,3], transcriptional downregulation [4], selective *HLA* allelic losses [5], hypermethylation [6] and loss of heterozygosity (LOH) involving *HLA* genes (LOH *HLA*) at chromosome 6 that causes *HLA* haplotype loss [7,8,9,10]. Frequently, the loss of one copy of the *B2M* gene due to LOH at chromosome 15 (LOH B2M), together with a mutation in the second allele, results in a complete loss of *HLA-I* expression [11,12,13]. All these mechanisms can be classified into regulatory (“soft”), which can be reversed by cytokines and structural (“hard”) defects producing irreversible *HLA* alterations [14]. Some alterations have higher incidence in certain types of malignancy. For instance, mutations in the *B2M* gene are typical for melanoma and MSI-H colorectal carcinoma [15], while a transcriptional downregulation of *HLA-I* can be frequently detected in bladder cancer [4]. LOH involving *HLA-I* genes is a frequent “hard” mechanism responsible for *HLA* haplotype loss that reduces the repertoire of tumor antigens presented to T-cells [13,16]. In normal conditions, all six *HLA-I* genes are expressed on each nucleated human cell. However, LOH can cause a simultaneous loss of all three *HLA-I* genes leading to a loss of *HLA* haplotype, or selective locus and allelic absences. It is known that each *HLA* allele can present a distinct range of tumor neoantigens to CTL. Thus, even a partial loss of *HLA-I* can impair specific antigen presentation and T-cell mediated tumor rejection. In a recent publication, loss of a single *HLA* locus or allele was shown to have an impact on the overall survival in cancer [17].

There is accumulating evidence that tumor *HLA-I* loss or altered expression reduces the efficacy of cancer immunotherapy and the mechanism is associated with the reduced T-cell cytotoxicity. More recently, LOH involving *HLA* genes has been linked to increased resistance to immunotherapy and cancer recurrence [18,19,20,21]. A subset of patients harboring LOH-6 in had poorer survival after treatment with checkpoint blockade therapy [22]. 

In general, LOH is a common form of allelic imbalance in cancer and the detection of LOH has been used to identify genomic regions that harbor tumor suppressor genes and to characterize different tumor types, pathological stages and progression. LOH can be caused by total chromosomal loss due to mitotic nondisjunction or by chromosomal deletions due to errors during mitotic recombination and a defective DNA damage response [23,24]. LOH of critical chromosomal regions in many cancers suggests a selection of LOH events that increase the survival of tumor cells during cancer initiation and progression, such as inactivation of tumor suppressor genes and immune genes [25]. Moreover, LOH could also be observed in premalignant cells and at early stages of neoplastic transformation. 

Therefore, it is important to characterize the pattern and extension of LOH-6 and -15, because LOH can cause a loss of the entire chromosomes, or only some parts that may or not include the *HLA* and *B2M* genes. In addition, there are chromosomal duplications observed during cancer progression that result in a copy number neutral LOH (CN-LOH), an alteration difficult to detect, which might potentially influence the ability of tumor cells to evade the antitumor immunity. We have previously reported in melanoma cell lines that the percentage of *HLA* homozygosity is much higher than in normal healthy donors [26]. In this case, a homozygous *HLA* genotype could suggest LOH at the *HLA* genetic region. Hence, it is important to develop techniques to detect LOH in the *HLA* and *B2M* genomic regions in DNA obtained from solid tumor tissues as well as in tumor cell lines.

In this study, using SNP arrays, we investigated the prevalence of LOH at chromosomes 6 and 15 in a number of human cancer cell lines of different histological type and discuss the potential immunologic and clinical implications of these chromosomal aberrations in tumor evolution.

## 2. Materials and Methods

### 2.1. Human Tumor Cell Lines and Tumors

We analyzed a total of 31 human tumor cell lines of distinct histological types (Table 1). Most of them, including 4 breast, 4 lung, 6 bladder cell lines and 4 myelodysplastic syndrome (MDS) blasts were randomly included into the study without previous knowledge of the *HLA* genotype. Bladder, breast and lung cancer cell lines were purchased from the American Type Culture Collection (ATCC). In addition, 13 melanoma cell lines were selected out of 91 in total (14%) based on a homozygous *HLA* genotype and positive total *HLA-I* cell surface expression [27]. 11 of them were from the European Searchable Tumor Cell Line Data Base (ESTDAB, http://www.ebi.ac.uk/ipd/estdab/, accessed on 5 November 2020 [26,27]. One melanoma cell line with total loss of *HLA-I* due to a *B2M* mutation (M010) was kindly provided by Dr. Gaudernak/Dr. Kyte (University of Oslo, Oslo, Norway) [13] and another melanoma cell line Ando-2 (with a loss of *HLA* haplotype) was kindly provided by Dr. Coulie (Université de Louvain (UCL), Brussels, Belgium). Malignant CD34+ blast cells were obtained from 27 patients with MDS from the Hospital Virgen de las Nieves in Granada, Spain [28]. Four of these patients showed LOH and these cases are included in Table 1.

Cell lines were cultured in Dulbecco’s Modified Eagle Medium (Sigma-Aldrich, St. Louis, MO, USA) or RPMI1640 supplemented with 10% FBS (GIBCO, Thermo Fisher Scientific, Waltham, MA, USA ), 2 mmol/L glutamine (Sigma-Aldrich, St. Louis, MO, USA) and 100 U/mL penicillin/streptomycin (GIBCO, Thermo Fisher Scientific, Waltham, MA, USA) at 37 °C in a 5% of CO_2_ atmosphere. 

In addition, we analyzed four bladder tumor tissue samples and autologous peripheral blood mononuclear cells (PBMCs) provided by the regional Biobank. Informed consent approved by the Ethics Committee of our institution was signed by all the patients included in this study. Previously, all medical records and tumor sections were reviewed by both urologist and pathologist. The tumor histological subtype in all cases was urothelial. These samples previously were studied by immunohistochemistry and showed positive *HLA-I*/*β2M* complex immunolabeling with selective *HLA* losses. 

### 2.2. Bladder Tumor Tissue Immunohistochemistry and Microdissection

After transurethral tumor resection bladder tumor tissue samples were immediately stored at −80 °C. Cryosections were obtained using a microtome-cryostat (Bright), allowed to dry at room temperature for 4–18 h, fixed in acetone at 4 °C for 10 min and stored at −80 °C until further analysis. Immunolabeling was performed using Biotin-Streptavidin kit (supersensitive Multilink HRP/DAB kit, BioGenex, The Hague, The Netherlands). Tissue sections were evaluated by two independent pathologists.

We analyzed the following molecules using specific monoclonal antibodies: W6/32-against *HLA-A*, *-B* and *-C* heavy chain/*β2M* complex (a gift from Dr. Bodmer, Imperial Cancer Research Fund Laboratories, London, UK); GRH-1, which recognizes free and heavy chain-associated *β2M* (produced and characterized in our laboratory); HC-10 against free *HLA-B* and *-C* heavy chains (Nordic-MUbio, Rangeerweg, The Netherlands), anti-*HLA-A* which recognizes a subset of *HLA-A* locus [29] and 42IB5 against the *HLA-B* locus [30]. 

For the LOH analysis we selected four tumor samples with positive *HLA-ABC*/*β2M* immunolabeling and selective loss of an *HLA* locus. Cryopreserved tissue sections (8–10 μm thick) from these four tumors were fixed in 70% ethanol, stained with a 0.05% *w*/*v* solution of toluidine blue and microdissected using a laser micromanipulator (PALM MicroLaser Systems, ZEISS Göttingen, Germany). The obtained fragments were collected in PALM Adhesive Caps and DNA was isolated from them using QIAamp Tissue Kit (QIAGEN, Hilden, Germany). In all cases, normal autologous DNA was obtained from PBMCs.

### 2.3. Analysis of HLA Genomic Copy Number Alterations by Single Nucleotide Polymorphism (SNP) Arrays 

**Figure 1:**
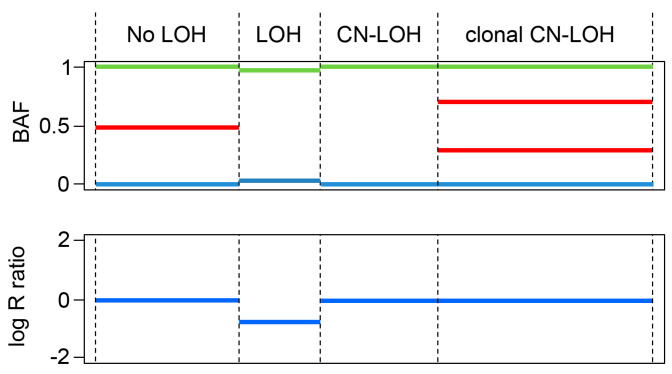
Schematic illustration of LOH assay using SNP arrays using the Illumina Infinium SNP Immunochip. The schematic genomic plots show patterns of B-allele frequency (BAF) (top panel) and corresponding log R ratios (bottom panel) along the analyzed chromosome (x-axis) demonstrating cases of heterozygosity (no LOH), LOH due to loss of genetic material (LOH), copy number neutral (CN-LOH) and clonal CN-LOH (a mixture of cells with CN-LOH and normal heterozygous cells).

DNA was isolated from the studied tumor cells and from microdissected bladder tumors and autologous PBMCs and was genotyped using the Illumina Infinium assay on the Immunochip according to manufacturer protocol. It detects about 200,000 SNPs selected based on GWAS of the diseases of the immune system. Loss of heterozygosity (LOH) and copy number results were obtained using the Illumina Genome studio software as “theta” and “R” values. The SNP analysis in each type of cells or tumors has been done twice with 100% reproducibility. We used immunochip data from unrelated samples of European ancestry to obtain a median fluorescence value per probe to create such a standard and to subsequently obtain log R ratios [10,28]. A log R ratio distribution around zero can be regarded as copy neutral (CN), while chromosomal intervals of mainly positive (or negative) log Ratios can be interpreted as a gain or a loss of chromosomal DNA. Figure 1 summarizes different patterns of LOH that can be obtained using SNP gene arrays. Upper plot shows the variants of B allele frequency (BAF), which indicates the zygosity of each SNP: a physiological situation that comprises equal representation of AA, AB and BB genotypes, respectively, and displaying B allele frequencies of 0, 0.5 and 1. Zero is frequency of allele A, 1 is the frequency of allele B and 0.5 corresponds to the heterozygous AB version of the genome. When the BAF pattern has all three mentioned above values, the sample is heterozygous (AB) and there is no LOH. When it lacks polymorphisms at 0.5, the sample is homozygous and has LOH. 

The lower plot shows the log R ratio, which is a measure of SNP CN variation, with a normal value defined as 0, reflecting a copy number neutral loss of heterozygosity (CN-LOH).

Some samples can have clonal LOH, or have a heterogeneous composition, representing a mixture of tumor cells with CN-LOH and without LOH. Some of these cells reflect clonal tumor evolution with heterogeneous CN-LOH. In that case, the heterogeneous AB pattern has an additional line at BAF and the distance between these two lines (red lines on Figure 1) will reflect the approximate percentage of cells with CN-LOH in the tumor cell mixture. We used the University of California in Santa Cruz (UCSC, Santa Cruz, CA, USA) Genome Browser (http://genome.ucsc.edu/, accessed on 6 March 2021) to map and characterize the range of the missing regions in chromosomes 6 and 15 (GRCh38/hg38 Assembly).

## 3. Results

###  Analysis of LOH-6 and LOH-15 in Human Cancer Cell Lines

We demonstrate CN-LOH at chromosomes 6 and 15 in a variety of human tumor cell lines, in leukemia blasts and in human bladder tumors using SNP genomic analysis. 

**Figure 2:**
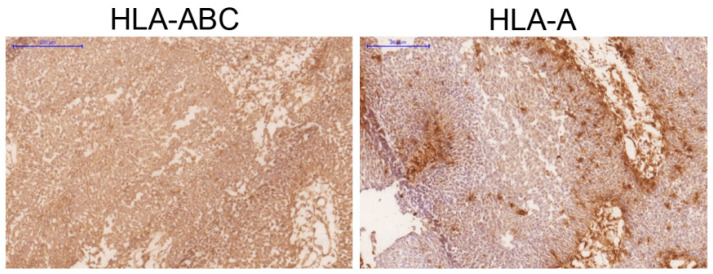
Immunohistological analysis of *HLA* class I expression in a bladder tumor tissue demonstrating a positive expression of total *HLA*-*ABC*/*β2M* complex and a selective loss of *HLA-A* locus. Blue scale bar: 200 μm.

**Figure 3:**
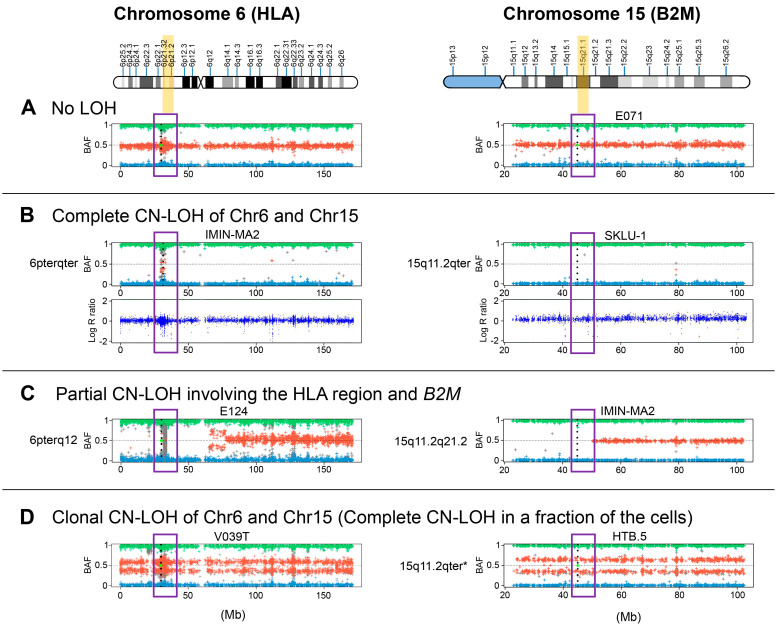
Examples of different CN-LOH patterns at chromosomes 6 (left panel) and 15 (right panel), involving *HLA* and *B2M* genomic regions, respectively, in human tumor cell lines and in microdissected bladder tumors. (**A**) Examples of cell lines without LOH; (**B**) Cell lines with a complete loss of a chromosome 6 and 15 (CN-LOH); (**C**) Partial CN-LOH involving *HLA* and *B2M* genes; (**D**) Complete CN-LOH in a fraction of cells, clonal CN-LOH (*).

Breast, lung and bladder cancer cell lines were selected randomly from the ATCC. Melanoma cell lines and bladder tumor tissue samples were selected based on the loss of *HLA* haplotype (homozygous *HLA* genotype) with apparently positive *HLA-ABC*/*β2M* expression detected by flow cytometry or immunohistochemistry with some selective locus or allelic loss [27]. Figure 2 depicts an example of a bladder tumor with a selective *HLA-A* locus loss. In the ESTDAB melanoma collection, around 14% (13/91) of the cell lines are homozygous for *HLA-I* [26,27]. We have previously observed an increased incidence of *HLA-I* homozygosity as compared to the control group (922 samples from the Spanish Bone Marrow Donor Registry) in which the frequency of homozygosity for *HLA-I* and *-II* genes is around 1.3% [28]. We detected CN-LOH involving the *HLA* region, with different deletions and complex patterns of chromosomal loss in all four leukemia blasts and in many cells lines: in two out of four breast cancer cell lines, in all four lung cancer cell lines and in three out of six bladder cancer cell lines (Table 1). Later we checked the *HLA* genotype of these cell lines and discovered that those with LOH *HLA* were homozygous, which confirms that LOH is a mechanism of *HLA* haplotype loss in these samples. Using SPN array assay we were able to see the extent of chromosomal alterations. Figure 3 demonstrates representative examples of observed LOH patterns in the studied tumor cell lines and tumors. Notably, 13 melanoma cell lines selected (out total of 91) for this study demonstrated different patterns of B-allelic Frequency indicating CN-LOH at chromosome 6 (14%) (Table 1). Among them, 7 cell lines with total loss of entire chromosome 6 (CN-LOH range-6pterqter) (Figure 3) showed a chromosome duplication and CN-LOH in chromosome 6 involving the region of the *HLA-A*, *HLA-B* and *HLA-C* genes (CN-LOH *HLA*).

We observed these patterns of CN-LOH in the majority of the studied cell lines, including the breast cancer cell lines MDA-MB4-435S and IMIM-MA2. On the contrary, MCF7 cells showed clonal CN-LOH in the region which does not include *HLA* genes (Table 1). SNP analysis of chromosome 15 showed different variations of LOH-15 in three breast cancer cell lines, CN-LOH in all IMIM-MA2 cells and clonal CN-LOH in MDA-MB4-435S and MCF7 cells. Notably, IMIM-MA2 showed CN-LOH involving both *HLA* genes and *B2M* (Figure 3). Overall, LOH-6 was more frequent than LOH-15 and without any correlation between these two alterations. 

Three out of six studied bladder cancer cell lines (selected randomly from ATCC) showed CN-LOH of the entire chromosome 6 (Table 1). The cell lines HTB2, 5637 and HTB5, presented CN-LOH only in 6q region without any alterations in *HLA-I* region. Almost all bladder cell lines also demonstrated CN-LOH at chromosome 15 and in four cell lines it involved the *B2M* gene region (Table 1). Importantly, all bladder cancer cell lines showed positive cell surface *HLA-I* /*β2M* expression as measured by FACs using a wide panel of antibodies against different *HLA* specificities. In some cases, there was a downregulation of *HLA-I* locus or allele, in most of the cases inducible by IFN-γ.

All four studied bladder tumors had CN-LOH at chromosome 6, in three of which this aberration involved *HLA-ABC* genes. In one of these three tumors, LOH-6 affected the entire chromosome 6 (6pterqter) (V039), but only in a proportion of cells (clonal CN-LOH-6 (Table 1, Figure 3). Tumor sample V24 also showed clonal CN-LOH in 6p region where *HLA* gene are located, but without a deletion of the entire chromosome (Table 1). Interestingly, three tumors with LOH-6 had no LOH-15 and the only LOH-6-free tumor V315 showed CN-LOH-15 involving entire chromosome 15 including the *B2M* gene.

Overall, a total loss of chromosome 6 (6pterqter) we found in 17 analyzed samples (97%), in two of which only a proportion of cells were affected by this alteration (clonal CN-LOH). Analysis of the region of chromosome 15 where the *B2M* gene is located, overall, demonstrated lower percentage of CN-LOH (54% or 19/31), of which in 15 cases it involved the *B2M* gene. Among these 15 samples, 12 showed a total loss of chromosome 15 (15q11.2qter) in all cells and in 10 cases this loss was detected only in a proportion of the cells (clonal LOH-15) (Table 1, Figure 3). In three samples, LOH-15 did not affect the *B2M* gene. Some examples of these patters can be seen in Figure 3. Thus, the total loss of chromosome 15 (15q11.2qter) was less frequent than the total LOH-6 (6pterqter) and it was mostly with a subclonal pattern. Notably, none of the CD34+ blasts from four patients with MDS showed LOH-15 (Table 1) and only one bladder tumor was with CN-LOH-15. 

In summary, the most relevant finding in our study is that LOH is a common chromosomal alteration in tumor cells and tissues. All the samples evaluated in this study (except for one) showed CN-LOH at chromosome 6 and more than half of the samples demonstrated CN-LOH at chromosome 15. Importantly, the most frequently observed pattern was CN-LOH-6 involving the whole chromosome 6 (6pterqter) (Table 1, Figure 3). 

## 4. Discussion

*HLA-I* is a highly polymorphic set of genes with each allele capable of presenting a specific and limited set of antigens to T cells. Humans are normally heterozygous at the *HLA*-*A,* -*B* and -*C* loci, which maximizes the variety of peptide that can be presented, improving responses to pathogens and tumors alike [31,32]. Hence, LOH-6 at *HLA* locus reduces the capacity of neoantigen presentation, thereby helping immune evasion of tumors. Importantly, even the loss of one *HLA* locus or allele can have an impact on the overall survival in cancer [17]. LOH-6 involving *HLA* genetic region is frequently found (20–40%) in a variety of tumors suggesting is a widespread mechanism occurring independently of the origin of the tumor, i.e., tissue or etiology [16]. In this study, we found LOH-6 in about 97% of the analyzed samples, most of them copy neutral (CN-LOH), involving the entire chromosome and causing *HLA* haplotype loss. The frequency of LOH-15 was lower (54%) and also mostly copy neutral (CN-LOH).

LOH causing *HLA* alterations might potentially influence the ability of tumor cells to evade the antitumor immunity, promote cancer progression and reduce patient survival [33]. In adult glioblastoma patients, LOH of *HLA-I* was associated with shorter overall survival [34]. In cervical carcinoma, LOH on 6p21.2 was found to be an important predictor of recurrence after radiation treatment, both overall survival and relapse-free survival were significantly worse for the patients with LOH as compared with those without LOH [35]. Previously, some groups have correlated loss of *HLA-I* with tumor grade/stage [36]. On the other hand, our group described LOH-6 in different types of cancer and did not see any link with tumor stage or differentiation grade [5]. In a more recent study of NSCLC, we did not find a direct association between tumor stage/grade and *HLA-I* or PD-L1 expression analyzed separately [10]. 

LOH-15 in *B2M* genetic area also is a frequent alteration in malignant cells [16]. It is enriched in different types of cancer, including breast, bladder and MSS colon carcinomas and it might be the initial event toward complete loss of the *B2M* gene. We have previously reported that LOH affecting *HLA* genes is an early event during carcinogenesis that is later followed by other genomic alterations such as *B2M* point mutations [13]. We were able to observe an immune escape of tumor cells harboring a specific *B2M* mutation in a primary tumor to a totally *HLA-I* negative tumor cells in a distant metastasis with the same mutation and LOH at chromosomes 15 and 6. Another study by Chen et al., also suggest that LOH in *HLA* occurs early on in tumorigenesis [37]. 

**Figure 4:**
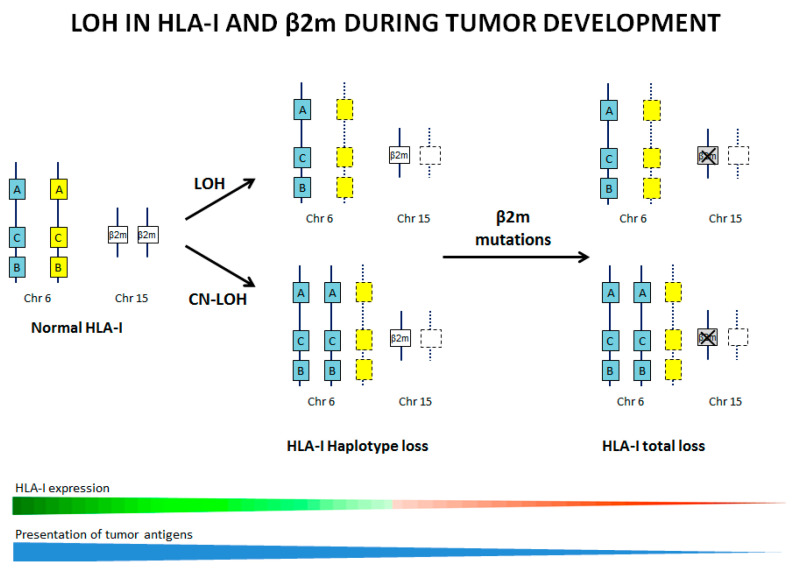
Schematic illustration of the accumulation of genetic alterations in chromosomes 6 and 15 (LOH, CN-LOH and *β2-microglobuline* mutations) accompanied by the decrease in tumor *HLA-I* expression and antigen presentation during the natural history of tumor development.

In this study, some samples showed a subclonal pattern of LOH, indicating the presence of a heterogeneous population of tumor cells within the sample, with and without LOH. Recent publications demonstrate a clonal evolution of tumors with LOH-6, involving the *HLA* genes, suggesting that this alteration is an early event in carcinogenesis and combined with other alterations, such as mutations in *β2M* or other genes involved in antigen presentation, could drive tumor evolution towards a more advanced stage. Importantly, there is strong evidence indicating that these *HLA* alterations accumulate as a result of a strong selective pressure from the tumor immune microenvironment leading to cancer immune escape and to resistance to immunotherapy. In Figure 4 we illustrate this hypothetical progressive accumulation of LOH and mutations in *HLA* genes during natural cancer evolution. Therefore, LOH involving the *HLA* genetic region can occur at very early stages of carcinogenesis, when tumor cells are still *HLA-I* positive.

Thus, while a strong anti-tumoral immune response is pivotal for the eradication of malignant cells, it can also promote escape variants by directly enhancing genomic instability (mutations/LOH in *HLA* and APM genes).

Understanding the primary and secondary immune evasion and discovering biomarkers that can predict the response to immunotherapies are of key importance. It seems that *HLA-I* homozygosity and LOH at *HLA-I* represent a genetic barrier to effective immunotherapy. Chowell et al., analyzed *HLA-I* genotype (*HLA-A*, *-B* and *-C*) in solid tumors from a cohort of cancer patients (NSCLC and melanoma) treated with anti-CTLA-4 or anti-PD-1 therapy and discovered that maximal heterozygosity at *HLA-I* loci improved overall survival after ICB compared with patients who were homozygous for at least one *HLA* locus [17].

Apart from being a predictive marker for the success of immunotherapy, LOH in *HLA* could result in secondary immune evasion which has been described in several cancers, including bladder, melanoma and lung cancer [38]. Studies by our laboratory showed that Bacillus Calmette-Guerin immunotherapy of bladder cancer induced the selection of *HLA-I*-deficient tumor cells with LOH in chromosome 6 and 15 [18]. Alterations in the *HLA-I* antigen presenting pathway were also detected in recurrent metastatic melanoma following C-Vax/BCG vaccine immunotherapy [39]. In a melanoma patient, resistance to peptide-based immunotherapy was linked to inactivation of *β2M* caused by a mutation and LOH at chromosome 15 [40]. Similarly, melanoma patients, receiving anti–PD-1 therapy (pembrolizumab), developed immune escape lesions with LOH in *JAK2* and a truncating mutation in *B2M* [19]. Sade-Feldman and colleagues analyzed escape lesions from five melanoma patients treated with Ipilimumab (anti-CTLA4 Abs) and found LOH in *B2M* in 29.4% of the cases [21]. Another example of acquire resistance to ICIs was described by Gettinger et al. who demonstrated a complete genomic loss of *B2M* (a copy number variation) in lung cancer lesions after anti-PD-L1 and anti-CTLA-4 treatment [20]. In addition, it has been reported that LOH on 6p21.2 correlates with the recurrence of cervical carcinoma after radiotherapy [35]. Therefore, detection and monitoring the evolution of tumor *HLA-I* expression defects before and during the course of treatment is also important to guide a selection of optimal immunotherapy. 

As we mentioned earlier, LOH is caused by a variety of genetic mechanisms. Our study shows that the most frequent mechanism is CN-LOH-6 (6pterqter) and CN-LOH-15 (15q11.2qter). In a recent publication, it was demonstrated that a whole-genome doubling (WDG) involving the duplication of a complete set of chromosomes, is a common feature in carcinogenesis [41]. WGD has been linked to increased tumor cell diversity, accelerated cancer genome evolution and worse prognosis [1,3,4,41]. In this sense, many observations suggest that LOH-6 and LOH-15 may be a far more prevalent form of immune evasion. Given the frequency of LOH detected in various types of untreated tumors, it may be important to analyze LOH-6 and -15 when designing patient-specific immunotherapy approaches. Importantly, this type of *HLA* alteration can be undetected and unnoticed in tumor cell or tissue with *HLA-I*/*β2M* positive surface expression. In Figure 4, we illustrate this progressive accumulation of LOH and mutations in *HLA* genes during natural cancer evolution. In our study, we found an increased incidence of CN-LOH involving *HLA* genes across different tumor cell types, highlighting the importance of accurate LOH detection as a pan-cancer biomarker. 

## 5. Conclusions 

LOH in the *HLA* locus is increasingly being recognized as an early and important mechanism of immune escape as well as a proposed biomarker for immunotherapy response. Neoantigens that bind to a deleted *HLA* allele will no longer be presented to the immune system, potentially allowing subclones with these deletions to escape immune surveillance. In this work, using SNP array analysis, we demonstrated that LOH-6 and -15 represent a common chromosomal alteration in tumor cells and tumor tissues of distinct histological type that are homozygous for *HLA* or show a selective loss of *HLA* expression. The incidence of LOH-6 was much higher than of LOH-15. Importantly, the most frequently observed pattern was CN-LOH-6 involving the whole chromosome 6 and duplication of the other one. Analysis of LOH in *HLA* genes may have important implications for the understanding a clonal evolution of tumors, for predicting response to immunotherapy and for the design of more efficient neoantigen-based therapeutic vaccines.

## Figures and Tables

**Table 1:** Analysis of the LOH in chromosomes 6 and 15 in human tumor cell lines and bladder tumors.

Histological Type	Sample	SNP-A Chr6—Range	Size (Mb)	LOH of the *HLA* Region	SNP-A Chr15—Range	Size (Mb)	LOH of the *B2M* Gene
*Melanoma (CCL)*							
	E037	CN-LOH 6pterqter		YES	CN-LOH 15q11.2qter *		YES
	E040	CN-LOH6pterp12.1,6pterq24.3 *	55, 148 *	YES	normal		NO
	E055	CN-LOH 6pterqter *,6q12qter	104	YES	normal		NO
	E058	CN-LOH 6pterqter		YES	CN-LOH 15q22.2qter *	41.8 *	NO
	E062	CN-LOH 6pterqter		YES	CN-LOH 15q11.2q23 *	50 *	YES
	E064	CN-LOH 6pterqter *		YES	normal		NO
	E070	CN-LOH 6pterp21.3	33.4	YES	normal		NO
	E071	CN-LOH 6pterqter		YES	normal		NO
	E081	CN-LOH 6pterqter		YES	normal		NO
	E019	CN-LOH 6pterqter *,6q15.1qter	177	YES	CN-LOH 15q14q22.2	25	YES
	E124	CN-LOH 6pterq12	65	YES	CN-LOH 15q11.2qter *		YES
	M010	CN-LOH 6pterqter		YES	CN-LOH 15q11.2qter		YES
	Ando	CN-LOH 6pterqter		YES	CN-LOH 15q11.2qter*		YES
*Breast (CCL)*							
	MDA-MB4-435	CN-LOH 6pterqter		YES	CN-LOH 15q21.1q21.3 *,15q22.2qter *	10 *,41 *	NO
	IMIM-MA2	CN-LOH 6pterqter		YES	CN-LOH 15q11.2q21.2	30	YES
	MCF-7	CN-LOH 6p21.31q25.2 *	122 *	NO	CN LOH 15q11.2q23 *	50 *	YES
	MDA-MB-231	Normal		NO	No data		No data
*Leukemia (CCL)*							
	X31364	CN-LOH 6pterp21.31	34	YES	normal		NO
	X28751	CN-LOH 6pterp12.3	47	YES	normal		NO
	X29708	CN-LOH 6pterp21.31	36	YES	normal		NO
	X30279	CN-LOH 6pterp21.2	38	YES	normal		NO
*Lung (CCL)*							
	SKMES	CN-LOH 6pterqter *,6pterp12.2	52	YES	CN-LOH 15q11.2qter *		YES
	SKLU-1	CN-LOH 6pterqter		YES	CN-LOH 15q11.2qter		YES
	CALU6	CN-LOH 6pterqter		YES	CN-LOH 15q11.2qter *		YES
	A427	CN-LOH 6pterqter		YES	normal		NO
*Bladder (CCL)*							
	RT4 (HTB-2)	CN-LOH 6q21q27 *	49	NO	CN-LOH 15q11.2qter *		YES
	5637 (HTB-9)	CN-LOH 6q16.3qter *	66 *	NO	CN-LOH 15q11.2qter *		YES
	TCCSUP (HTB-5)	CN-LOH 6pterp22.3,6q12q25.2	22, 87	NO	CN-LOH 15q11.2qter *		YES
	WILL	CN-LOH 6pterqter		YES	CN-LOH 15q11.2q14 *,15q21.3q26.2 *	16 *, 42 *	NO
	J82 (HTB-1)	CN-LOH 6pterqter		YES	normal		NO
	T24 (HTB-4)	CN-LOH 6pterqter		YES	CN-LOH 15q11.2qter *		YES
*Bladder (TT)*							
	V039	CN-LOH 6pterqter *		YES	normal		NO
	V164	CN-LOH 6pterp21.1	49.5	YES	normal		NO
	V24	CN-LOH 6pterp21.2	39	YES	normal		NO
	V315	CN-LOH 6q13qter	95.1	NO	CN-LOH 15q11.2qter		YES

*CCL*: cancer cell lines, *TT*: tumor tissue, * Heterogeneous or clonal CN-LOH, with only a percentage of affected cells.

## Data Availability

The data presented in this study is contained within the article and are available on request from the corresponding author.
